# Ticagrelor: The First Reversibly Binding Oral P2Y_12_ Receptor Antagonist

**DOI:** 10.1111/j.1755-5922.2009.00096.x

**Published:** 2009

**Authors:** Steen Husted, JJJ van Giezen

**Affiliations:** 1 Department of Medicine and Cardiology, Aarhus University HospitalAarhus, Denmark; 2 AstraZenecaMölndal, Sweden

**Keywords:** Ticagrelor, AZD6140, platelets, inhibition of platelet aggregation, P2Y_12_ receptor, reversibility

## Abstract

Ticagrelor (AZD6140) is the first reversibly binding oral P2Y_12_ receptor antagonist that blocks ADP-induced platelet aggregation. Unlike thienopyridines, which irreversibly bind to the P2Y_12_ receptor for the lifetime of the platelet, ticagrelor binds reversibly to the receptor and exhibits rapid onset and offset of effect, which closely follow drug exposure levels. Animal models indicate greater separation between antithrombotic effects and bleeding effects with ticagrelor than with thienopyridines. Unlike the thienopyridines, ticagrelor does not require metabolic activation. It is quickly absorbed and exhibits a rapid antiplatelet effect, with higher and more consistent levels of inhibition of platelet aggregation (IPA) being maintained across the dosing interval than with clopidogrel. IPA levels decline with plasma drug levels after discontinuation of dosing. In the phase II DISPERSE-2 trial of 990 patients with non-ST-elevation acute coronary syndromes (ACS), ticagrelor treatment with 90 mg and 180 mg twice daily showed comparable rates of major and minor bleeding compared with clopidogrel 75 mg while there were numerically fewer myocardial infarctions. Ticagrelor resulted in greater IPA in clopidogrel-naïve patients and produced substantial additional reductions in platelet aggregation activity in patients pretreated with clopidogrel. Ticagrelor treatment was well tolerated in DISPERSE-2, and discontinuation rates were comparable to those observed for clopidogrel. An increased risk of mild to moderate dyspnea and mostly asymptomatic ventricular pauses were observed in phase II studies. The mechanisms for these effects are currently being investigated. The efficacy and safety of ticagrelor are being further evaluated in the phase III PLATO trial, involving approximately 18,000 patients with ACS, including both ST-elevation and non-ST-elevation ACS.

## Introduction

Oral antiplatelet therapy including a platelet P2Y_12_ receptor inhibitor is a cornerstone of antithrombotic treatment in patients with acute coronary syndromes (ACS) with or without ST-segment elevation and in patients undergoing percutaneous coronary intervention (PCI) [1–4]. However, the thienopyridine clopidogrel, the most widely used P2Y_12_ inhibitor, has a number of important limitations. First, the irreversible inhibition of platelets (a thienopyridine characteristic) that persists throughout the lifetime of the platelet may complicate management in patients who might require surgery and would therefore be at increased risk of bleeding. Second, clopidogrel requires hepatic conversion to an active metabolite (a thienopyridine characteristic), resulting in delayed onset of effect and posing the potential for variable interindividual platelet effects associated with variable metabolism [5–8]. Third, mean levels of inhibition of adenosine diphosphate (ADP)-induced platelet aggregation observed with clopidogrel are modest and responses to this agent are variable [9–14], including hyporesponsiveness that has been associated with increased risk of adverse clinical outcomes [15–25]. These drawbacks may be partly overcome with prasugrel, a new thienopyridine agent that is more efficiently metabolized to its active form and whose magnitude and consistency of platelet ADP inhibition is greater than clopidogrel [26–28].

Results from the TRITON-TIMI 38 study (Trial to Assess Improvement in Therapeutic Outcomes by Optimizing Platelet Inhibition with Prasugrel-Thrombolysis in Myocardial Infarction 38) have shown that prasugrel treatment compared with clopidogrel in moderate-to-high-risk ACS patients scheduled for PCI significantly reduced occurrence of the primary efficacy endpoint of death from cardiovascular causes, nonfatal myocardial infarction, or nonfatal stroke (9.9% vs. 12.1%; hazard ratio [HR] 0.81; 95% CI 0.73–0.90). However, the improved clinical benefits were accompanied by a significantly increased risk for non-CABG-related TIMI major bleeding, occurring in 2.4% of patients treated with prasugrel and 1.8% of patients treated with clopidogrel (HR 1.32; 95% CI 1.03–1.68) [29].

Ticagrelor (AZD6140), the first reversibly binding oral P2Y_12_ receptor antagonist, has the potential to address many of the limitations of thienopyridine therapy. Here, we review evidence demonstrating that ticagrelor (1) is not a prodrug and therefore does not require metabolic activation, has a rapid and reversible concentration-dependent inhibitory effect on the P2Y_12_ receptor, and appears to offer a wider separation between antithrombotic effects and bleeding time in preclinical models; (2) provides greater and more consistent inhibition of ADP-induced platelet aggregation than clopidogrel without an increase in major plus minor bleeding in phase II studies; (3) offers the potential for greater flexibility in the management of patients at risk for thrombotic events due to rapid onset and offset of antiplatelet effect; and (4) may also exert antithrombotic activity beyond platelet inhibition by inhibiting P2Y_12_-mediated vasoconstriction in vascular smooth muscles.

## Chemistry

**Figure 1:**
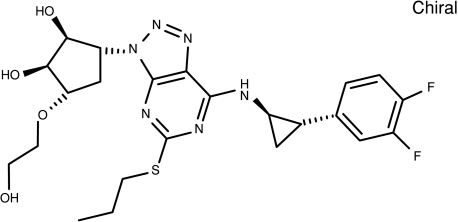
Chemical structure of ticagrelor. Adapted with permission from van Giezen and Humphries [30].

Ticagrelor [(1*S*,2*S*,3*R*,5*S*)-3-[7-{[(1*R*,2*S*)-2-(3,4-difluoro-phenyl)cyclopropyl]amino}-5-(propylthio)-3*H*-[1,2,3]-triazolo[4,5-*d*]pyrimidin-3-yl]-5-(2-hydroxyethoxy)cyclopentane-1,2-diol] is a cyclopentyl-triazolo-pyrimidine (CPTP), a new chemical class of antiplatelet agents that differs from both thienopyridines and ATP analogs (Figure 1) [30,31]. Discovery of this class arose from efforts to identify stable analogs of ATP, the natural antagonist of the P2Y_12_ receptor, to be used as a pharmacologic tool for study of P2Y_12_ activity (Table 1) [31]. Exploration of structure–activity relationships showed affinity-increasing propertie of substituents in the 2 position of the ATP adenine ring and stability-increasing properties of β,γ-methylene substitutions in the triphosphate. Beginning with the resultant ATP analogs, a drug development program led to identification of several potent selective P2Y_12_ antagonists, which, due to the short half-life resulting from retention of the triphosphate chain, would require intravenous (IV) administration. One of these compounds, the ATP analog cangrelor (AR-C69931MX), is being developed as an IV antiplatelet agent. Subsequent modifications to these compounds included elimination of phosphates and changes in the core purine and sugar moiety, resulting in identification of the first selective and stable nonphosphate P2Y_12_ antagonist AR-C109318XX. Further refinement to improve oral bioavailability resulted in development of ticagrelor, the first CPTP to be developed clinically.

**Table 1:** Key elements in the medicinal, chemical journey from ATP through the ATP analog cangrelor to the CPTP ticagrelor

• Introducing affinity-enhancing 5,7-hydrophobic substituents
• Replacing the labile triphosphates group
• Changing the core purine to a triazolopyrimidine, increasing affinity 100-fold
• Finding the first nonacidic reversible antagonists
• Introducing the *trans*-2-phenylcyclopropylamino substituent, increasing affinity more than 10-fold
• Identifying metabolically stable neutral compounds by modifying the hydrophobic phenylcyclopropyl group and the hydroxylic side chain substituent

From Springthorpe et al. [31].

## Preclinical Pharmacology

Binding studies in rh-P2Y_12_ receptor-transfected CHO-K1 cells indicate that ticagrelor exhibits potent, rapid, and reversible binding, with a K_d_ of 10.5 nM, a k_on_ (association constant) of 0.00011/(nM·s), a k_off_ (dissociation constant) of 0.00087/s, and half-life values of 4 min for binding and 14 min for unbinding [32]. The rapid on/off receptor kinetics of ticagrelor indicate that its inhibitory effect closely reflects levels of drug exposure.

The selectivity of effect for the P2Y_12_ receptor was shown in a murine laser-injury model, in which ticagrelor reduced thrombus formation in P2Y_12_-positive mice (+/+) to levels found in P2Y_12_-deleted mice (−/−), with no additional suppression of thrombus formation observed in the latter with ticagrelor treatment [33].

**Figure 2:**
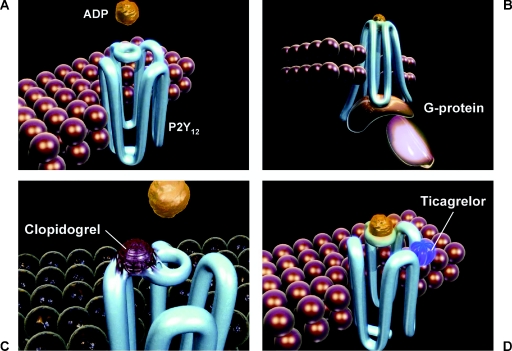
(**A** and **B**) ADP binds to the P2Y_12_ receptor, resulting in conformational change and G-protein activation. (**C**) Binding of the clopidogrel active metabolite to the P2Y_12_ receptor is irreversible, rendering the receptor nonfunctional for the life of the platelet. (**D**) Ticagrelor binds reversibly to P2Y_12_ at a site distinct from the ADP-binding site and inhibits ADP signaling and receptor conformational change by “locking” the receptor in an inactive state; the receptor is functional after dissociation of the ticagrelor molecule. ADP can still bind at its binding site, and the degree of receptor inhibition (and inhibition of ADP-induced signaling) is dependent on the concentration of ticagrelor. Adapted with permission from van Giezen [36].

Ticagrelor binds to the P2Y_12_ receptor at a site distinct from the ADP-binding site, as initially shown in studies in which CPTP did not prevent binding of ADP [34], and appears to inhibit ADP-induced receptor signaling in a noncompetitive manner [35]. In studies in rh-P2Y_12_-transfected CHO-K1 cells using a GTP_γ_S G-protein binding assay, ADP acted as a partial agonist of P2Y_12_ compared with 2-MeSADP (150-fold greater potency and 2.5-fold greater binding effectiveness), a potent P2Y_12_ agonist that interfered with binding of both ADP and ticagrelor. Ticagrelor competitively inhibited 2-MeSADP-induced receptor signaling, displacing the concentration–response curve to the right, and noncompetitively inhibited ADP-induced signaling, as indicated by a right-shifting of the concentration–response curve and by suppression of the ADP-induced signal (reduction of E_max_). Since ticagrelor does not prevent ADP binding, it is hypothesized that it acts by inhibiting the receptor conformational change and G-protein activation induced by ADP binding, hence “locking” the receptor in an inactive state and inhibiting ADP signaling (Figure 2) [36]. Unlike thienopyridines, which bind covalently to P2Y_12_, induce conformational change, and render the receptor permanently inactivated, ticagrelor binding leaves the receptor intact upon dissociation. Thus, inhibition of platelet aggregation (IPA) is dependent on the concentration of drug in plasma available to occupy receptors. Offset of inhibitory effect should reflect plasma drug levels with cessation of effect closely following drug administration/availability and degree of IPA dropping with declining systemic drug concentrations.

The greater affinity for the P2Y_12_ receptor and greater potency in platelet inhibition for ticagrelor than those of prasugrel were demonstrated in *in vitro* studies with compound 105, a chemical compound indistinguishable from the active metabolite of prasugrel [37]. In a P2Y_12_ receptor filtration assay, ticagrelor exhibited up to 100 times the comparator's affinity for P2Y_12_ (on measurement of 50% inhibitory concentration, IC_50_) at shorter incubation time to 1 min (IC_50_ of 0.074 ± 0.038 μM for ticagrelor vs. 8.27 ± 2.91 μM for compound 105) and reached equilibrium within 15 min. In comparison, no equilibrium was reached with compound 105 over the 2-h measurement period (by IC_50_ and ^125^I-285 displacement), indicating slow receptor kinetics for the latter. Ticagrelor exhibited 48-fold greater potency in inhibiting 2-MeSADP receptor activation in the GTP_γ_S binding assay compared with compound 105 (IC_50_ of 0.059 ± 0.03 μM vs. 2.84 ± 0.83 μM) and 63-fold greater potency in inhibiting platelet aggregation in an ADP-induced washed-platelet aggregation assay (IC_50_ of 0.005 ± 0.004 μM vs. 0.313 ± 0.12 μM). These findings support the rapid onset of antiplatelet effect and high levels of platelet inhibition observed with ticagrelor in clinical evaluation.

**Figure 3:**
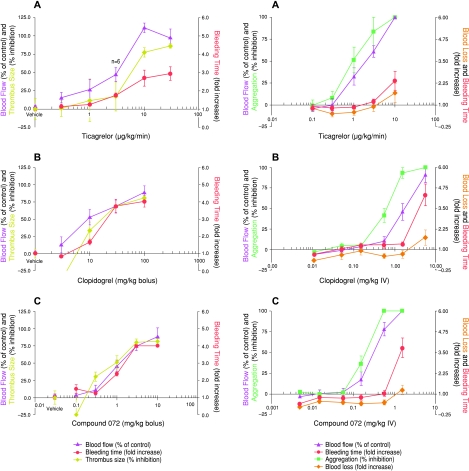
Antithrombotic effects and bleeding time with (**A**) ticagrelor, (**B**) clopidogrel, and (**C**) compound 072, a chemical compound indistinguishable from prasugrel, in animal models. *Left panels:* Percent of thrombus size inhibition, percent of arterial blood flow, and n-fold increase in bleeding time versus control according to dose of each drug in anesthetized rat model. Data are mean ± standard error of the mean (SEM); n = 4–6. *Right panels:* Percent of arterial blood flow, percent of IPA *ex vivo*, and n-fold increase in blood loss and bleeding time versus control according to dose of each drug in anesthetized dogs. Data are mean ± SEM; n = 6. Adapted with permission from van Giezen et al. [38].

The binding characteristics of ticagrelor appear to be associated with a wider separation between antithrombotic effects and bleeding effects than that seen with thienopyridines, as shown in rat and dog models of hemostasis and thrombosis [30,38]. In both the dog cyclic flow reduction model (modified Folts model) and a rat model using FeCl_3_ intimal injury, IV ticagrelor, clopidogrel, and compound 072, a chemical compound indistinguishable from prasugrel, dose-dependently inhibited arterial thrombus formation. Results in both models suggested that ticagrelor was able to achieve higher levels of antithrombotic effects than thienopyridines, without an equivalent increase in associated bleeding time (Figure 3, Table 2). In the rat model, the separation between antithrombotic and bleeding effects was calculated as the ratio of the dose resulting in a 3.0-fold increase in tail bleeding time (ED_BT:3.0_) to the dose restoring blood flow to 50% of control values (50% effective dose, ED_50_). This ratio (ED_BT:3.0_/ED_50_) was 9.7 with ticagrelor, compared with 2.0 with clopidogrel and 1.4 with compound 072. In dogs, the ratio for the dose resulting in a 3.5-fold increase in tongue bleeding time to the ED_50_ for blood flow (ED_BT:3.5_/ED_50_) was >5.2 with ticagrelor (bleeding time was not increased by 3.5-fold at the highest ticagrelor dose; see Figure 3) compared with 2.3 with clopidogrel and 4.3 with compound 072.

**Table 2:** Effects of ticagrelor, clopidogrel, and compound 072, a chemical compound indistinguishable from prasugrel, in rat and dog models of arterial thrombosis and hemostasis

	Ticagrelor	Clopidogrel	Compound 072
Rat model			
Blood flow (ED_50_)	3.1 μg/(kg·min)	10 mg/kg	1.1 mg/kg
Bleeding time (ED_BT:3.0_)	30 μg/(kg·min)	20 mg/kg	1.5 mg/kg
ED_BT:3.0_/ED_50_	9.7	2.0	1.4
Inhibition of thrombus size (ED_50_)	4.5 μg/(kg·min)	17 mg/kg	1.0 mg/kg
Dog model			
Blood flow (ED_50_)	1.90 μg/(kg·min)	1.68 mg/kg	0.3 mg/kg
Bleeding time (ED_BT:3.5_)	>10 μg/(kg·min)	3.9 mg/kg	1.3 mg/kg
ED_BT:3.5_/ED_50_	>5.2	2.3	4.3
*Ex vivo* platelet aggregation (ED_50_)	1.02 μg/(kg·min)	0.62 mg/kg	0.2 mg/kg

From van Giezen et al. [38].

ED_50_, dose yielding 50% antithrombotic effect; ED_BT_, dose yielding n-fold increase in bleeding time versus control.

Data are combined for all doses. Therapeutic window defined as the ratio of the dose inducing a 3.0-fold (rats) or 3.5-fold (dogs) increase in bleeding time to the dose inducing a 50% antithrombotic effect as measured via restoration of blood flow.

### Potential Effect on ADP-Induced Vasoconstriction

Preclinical studies also suggest that reversible P2Y_12_ inhibition may be associated with beneficial effects on P2Y_12_-mediated vasoconstriction, effects that may permit reduction in thrombogenic vasospasm or reduce deficits in myocardial perfusion after thrombosis. P2Y_12_ receptors are present in vascular smooth muscle cells in concentrations greater than other ADP receptors – for example, P2Y_1_ and P2Y_13_– and are active in stimulating vessel contraction. In studies in arterial segments from patients undergoing coronary bypass, P2Y_12_-mediated vasoconstriction was demonstrated using 2-MeSADP-induced contraction of submaximally precontracted vessels [39]. This contraction was blocked by a selective reversible P2Y_12_ antagonist (AR-C67085) and not blocked by a selective P2Y_1_ antagonist. No inhibition of contraction was observed in vessels from patients pretreated with clopidogrel. It is hypothesized that the short half-life and physicochemical properties of thienopyridine active metabolites prevent these drugs from entering the vascular wall and that systemically available drugs such as ticagrelor may, in contrast, be available to interact with these receptors.

The ability of ticagrelor to inhibit ADP-induced contractions in vascular smooth muscle was shown in an *ex vivo* study in denuded mouse aortic rings. In these studies, 2-MeSADP-induced contractions were 59% of maximal values (obtained with potassium-rich buffer) in mice that were not pretreated and 64% in mice pretreated with clopidogrel 50 mg/kg [40]; the addition of ticagrelor 10 μM to the vessel segment in tissue bath resulted in significant inhibition of ADP-induced contraction, to 33% of maximal in the untreated group and 32% of maximal in the clopidogrel-pretreated group. These findings suggest that ticagrelor could modulate vasoreactivity mediated by locally increased levels of ADP *in vivo*; pretreatment with clopidogrel does not appear to inhibit ADP-induced contractions. The potential effects of such inhibition were shown in a study in a dog thrombosis model, in which treatment with the selective P2Y_12_ antagonist AR-C69931MX (4 μg/(kg·min)) resulted in decreased reocclusion and cyclic flow variation and improved myocardial flow compared with placebo in animals receiving tissue-type plasminogen activator and heparin after thrombus formation [41]. Another study using the same thrombosis model in dogs compared adjunctive infusion of ticagrelor (75 μg/kg IV bolus, followed by 10 μg/(kg·min) IV infusion over 2 h) with clopidogrel (10 mg/kg IV bolus over 5 min) and placebo, on top of fibrinolytic therapy (t-PA + heparin). In this study, both clopidogrel and ticagrelor treatment resulted in complete blockade of ADP-induced platelet activation, aggregation, and recruitment, and prevented platelet-mediated thrombosis. However, despite similar antiplatelet effects, animals treated with ticagrelor had significantly lower rates of reocclusion (0% for ticagrelor vs. 30% for clopidogrel), less cyclic flow variation (0% vs. 30%), longer reflow duration (120 ± 0.0 min vs. 96.9 ± 38.9 min), and greater reductions in infarct sizes (final size of 6.31 ± 2.86 cm^2^ vs. 14.63 ± 4.29 cm^2^) [42]. Taken together, these data suggest that the systemic presence of reversible inhibitors may exert additional benefit, for example, via inhibition of nonplatelet P2Y_12_ receptors.

Ticagrelor may confer additional benefits in patients with ACS by inhibiting uptake of adenosine by human erythrocytes. In suspensions of washed human erythrocytes, ticagrelor dose-dependently inhibited adenosine uptake by erythrocytes with a pIC_50_ of 7.0 ± 0.1 (n = 5), which was 10-fold less potent than dipyridamole [43]. Adenosine is one of the mediators of the reactive hyperemia response to temporary coronary artery occlusion. *In vivo* data from a canine model of coronary blood flow regulation confirmed that both ticagrelor (n = 8) and dipyridamole (n = 8) dose-dependently augment the hyperemic response to temporary occlusion of direct intracoronary adenosine infusion, without affecting measured blood flow in the circumflex artery. In vehicle-treated animals, infusion of adenosine 15 μg/min or 30 μg/min resulted in blood flow in the left anterior descending artery 126.8 or 227.3% of baseline, respectively. Systemic administration of ticagrelor 30 or 100 μg/(kg·min) resulted in blood flow 114 ± 10% or 150 ± 19% of baseline, respectively, following infusion of adenosine 15 μg/min, and 109 ± 10% or 140 ± 13% of baseline, respectively, following infusion of adenosine 30 μg/min. Administration of dipyridamole 0.17 or 0.50 μg/(kg·min) resulted in blood flow 160 ± 2% or 193 ± 17% of baseline following adenosine 15 μg/min, and 136 ± 17% or 153 ± 30% following adenosine 30 μg/min. These results confirm that both ticagrelor and dipyridamole augment the adenosine-induced increase in coronary blood flow in this model and suggest that ticagrelor may deliver additional benefits to patients with ACS by enhancing adenosine-induced coronary blood flow increases. Translation of these results to clinical benefit will need to be confirmed with clinical data [43].

## Pharmacokinetics/Pharmacodynamics in Healthy Subjects

Ticagrelor is rapidly absorbed following oral administration. It is metabolized primarily via cytochrome P450 3A enzymes and has one known active metabolite, AR-C124910XX, that is present in blood at approximately one-third of the concentration of the parent drug [30]. Although this metabolite has potency in inhibiting the P2Y_12_ receptor equivalent to that of the parent compound, metabolic activation is not a requirement for IPA to occur. The pharmacokinetics of ticagrelor and its metabolite are predictable, with plasma concentrations being dose-proportional after initial dosing and stable at steady state [44].

In six subjects receiving a single oral dose of ^14^C-ticagrelor 200 mg suspension, the average total recovery of radioactive dose was 84.3%, consisting of 26.5% in urine and 57.8% in feces [44]. The mean radioactivity plasma/blood ratio was 1.69, indicating that most radioactivity is restricted to the plasma space. Ticagrelor and its active metabolite, AR-C124910XX, constituted the major components identified in feces, plasma, and less than 1% of components in urine. The primary components in urine were the inactive metabolite AR-C133913XX and its glucuronide conjugate. These findings indicate that ticagrelor and AR-C124910XX are extensively metabolized and suggest that renal impairment may have little effect on systemic exposure to the active compounds. No extensive penetration or binding to erythrocytes was observed for either ticagrelor or the active metabolite.

In single-dose pharmacokinetic/pharmacodynamic studies [46,47], subjects received oral ticagrelor in doses of 0.1–100 mg, 30–400 mg, or 900–1260 mg. Absorption was rapid (median t_max_ 1.5–3 h for both ticagrelor and its active metabolite), area under the concentration–time curve (AUC) and C_max_ increased in an apparently dose-proportional manner, and t_1/2_ was 7.1–12 h for ticagrelor (8.5–10.1 h for the active metabolite). At 2 h postdose, IPA (20 μM ADP) measured by optical aggregometry for final extent was 88–95% at doses of 100–400 mg; at these doses, IPA plateaued at 88–100% and decreased to 74–89% at 12 h. No safety/tolerability issues occurred at doses up to 900 mg, with dose-limiting gastrointestinal adverse events such as nausea, vomiting, and abdominal pain occurring at 1260 mg.

In multiple-dose studies in healthy volunteers, subjects (n = 48) received ticagrelor 30–600 mg once daily (qd) or 50–300 mg twice daily (bid) for 16–20 days or clopidogrel at a 300-mg loading dose plus 75 mg/day for 14 days [44,48]. Plasma levels of ticagrelor peaked 1.5–3 h after dosing and reached steady state after 2–3 days. Mean t_1/2_ ranged from 6.2 to 13.1 h with qd dosing and from 6.6 to 9.1 h with bid dosing, and C_max_ and AUC increased in a dose-proportional manner. IPA (20 μM ADP, final extent) closely followed ticagrelor plasma levels and was thus dose- and time-dependent. IPA was higher and less variable at ticagrelor doses of more than 100 mg bid and more than 300 mg qd than with clopidogrel 75 mg. At bid dosing of 100 mg, the profile of which is similar to the 90 mg formulation taken twice daily, or higher, IPA was nearly complete (>90%) over 24 h (Table 3). Bleeding time was modestly prolonged compared with placebo; from a median baseline value of 165 seconds, a 1.1- to 3.3-fold increase in bleeding time was seen after administration of ticagrelor versus a 1.1- to 1.2-fold increase with placebo. Treatment was well tolerated with no serious or dose-related adverse events.

**Table 3:** Mean IPA (20 μM ADP, final extent) over 24 h at specified testing times in healthy subjects receiving ticagrelor 50–300 mg bid, clopidogrel 300-mg loading dose or 75 mg/day, or placebo

	n	Inhibition (%), mean (range)			
Treatment group		4 h	8 h	12 h	24 h
Ticagrelor					
50 mg bid, day 1	14	92 (55–100)	82 (19–100)	66 (0–100)	88 (45–100)
50 mg bid, day 5	14	95 (62–100)	90 (27–100)	87 (13–100)	79 (3–100)
100 mg bid, day 5	13	97 (72–100)	95 (63–100)	93 (43–100)	93 (65–100)
200 mg bid, day 5	13	98 (85–100)	98 (89–100)	96 (79–100)	97 (76–100)
300 mg bid, day 5	7	100 (100–100)	100 (100–100)	99 (97–100)	100 (100–100)
Clopidogrel					
300-mg loading dose, day 1	14	67 (0–100)	52 (0–98)	57 (0–100)	56 (0–100)
75 mg, day 14	14	90 (35–100)	82 (14–100)	83 (30–100)	77 (11–100)
Placebo	39	7 (0–25)	8 (0–38)	8 (0–48)	5 (0–28)

From Peters et al. [48], Butler et al. [44].

Day refers to day within treatment group, not day within study.

A study in which healthy subjects were coadministered ticagrelor 50 or 200 mg bid and aspirin 300 mg qd for 10 days showed no effect of aspirin on ticagrelor pharmacokinetics, ADP-induced platelet aggregation response, or rate or extent of IPA [49]. However, aspirin increased the inhibition of collagen-induced platelet aggregation (e.g., from 20%[range 0–43%] with ticagrelor 200 mg alone to 76%[range 55–92%]), reflecting the combined antiplatelet effects of the two agents.

## Phase II Studies: Pharmacokinetics/Pharmacodynamics and Clinical Effects

Phase II studies have shown that ticagrelor treatment produces rapid, high, and consistent IPA, with degree of IPA reflecting plasma drug levels, with no increase in major plus minor or major bleeding rates compared with clopidogrel. Ticagrelor treatment was generally well tolerated in these studies. Safety findings included an apparent dose-related incidence of dyspnea, a greater incidence of mostly asymptomatic ventricular pauses in ticagrelor patients, and a mild increase in uric acid levels.

## DISPERSE: Patients with Stable Atherosclerosis

In the DISPERSE trial [50], 200 patients with stable atherosclerotic disease were randomized to ticagrelor 50 mg (n = 41), 100 mg (n = 39), or 200 mg (n = 37) bid or to 400 mg qd (n = 46) or to clopidogrel at the standard maintenance dose of 75 mg/day (n = 37) for 28 days along with aspirin (75–100 mg/day). The main pharmacodynamic measure was inhibition of ADP-induced platelet aggregation (20 μM ADP) measured by optical aggregometry, and the primary tolerability measure was the incidence of adverse events.

### Pharmacokinetics

Pharmacokinetic parameters are shown in Table 4[50]. Drug and metabolite concentrations increased in a linear and dose-proportional manner and were stable and predictable at steady state (reached by day 14). At day 28, pharmacokinetics were slightly more than dose-proportional in the 200 mg bid and 400 mg qd groups (dose-normalized AUCs approximately 50% more than dose proportional with correspondingly lower total plasma oral clearance than seen with lower doses). C_max_ and AUC values did not vary by gender or age 65 years or less or more than 65 years for ticagrelor.

**Table 4:** Mean (coefficient of variation percentage) pharmacokinetic values for ticagrelor and active metabolite in patients in DISPERSE

	50 mg bid		100 mg bid		200 mg bid		400 mg qd	
	Day 1 (n = 41)	Day 28 (n = 38)	Day 1 (n = 39)	Day 28 (n = 33)	Day 1 (n = 37)	Day 28 (n = 35)	Day 1 (n = 46)	Day 28 (n = 39)
Ticagrelor								
t_max_ (h)	3.66 (41)	3.33 (56)	3.05 (50)	2.52 (55)	3.09 (57)	2.74 (82)	2.03 (63)	2.12 (71)
C_max_ (ng/mL)	287 (70)	387 (57)	594 (55)	798 (59)	1224 (35)	2200 (41)	3374 (41)	3827 (42)
AUC (ng·h/mL)	1640 (50)	2688 (56)	3648 (56)	5337 (45)	7581 (35)	15,104 (39)	NA	31,338 (53)
CL/F (L/h)	NA	23.7 (48)	NA	22.6 (44)	NA	15.3 (39)	NA	15.6 (39)
AR-C124910XX								
t_max_ (h)	4.23 (33)	3.25 (61)	3.69 (32)	3.22 (45)	3.71 (43)	3.16 (69)	3.17 (41)	3.26 (53)
C_max_ (ng/mL)	73 (109)	118 (61)	135 (50)	239 (38)	271 (39)	660 (51)	595 (32)	860 (47)
AUC (ng·h/mL)	418 (58)	906 (48)	899 (46)	1881 (32)	1753 (32)	5268 (41)	–	10,466 (45)

From Husted et al. [50].

CL/F, total plasma oral clearance.

### Inhibition of Platelet Aggregation

**Figure 4:**
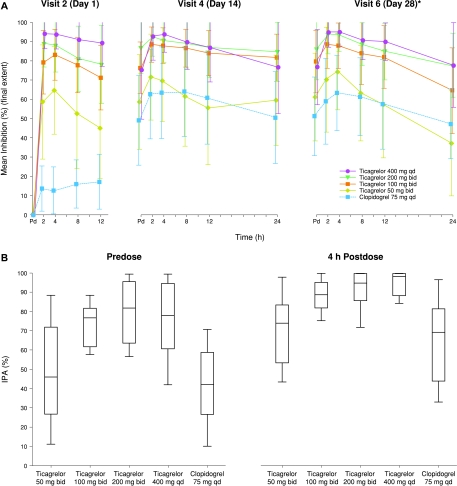
(**A**) Mean IPA (20 μM ADP, final extent) in patients in DISPERSE receiving ticagrelor 50, 100, or 200 mg bid or 400 mg qd or clopidogrel 75 qd on day 1, day 14, and day 28. *No second dose of ticagrelor was given on day 28. Error bars indicate standard deviation, shown only for IPA sample space. (**B**) Median (line in box), 25–75% percentile (box), and 10–90% percentile (whiskers) IPA predose and 4-h postdose on day 14 (final extent). Adapted with permission from Husted et al. [50].

Ticagrelor exhibited peak IPA (final extent) at 2- to 4-h postdose on day 1 and at steady state (day 28), whereas clopidogrel minimally inhibited aggregation on day 1 (<20% at all time points). The 100-mg and 200-mg bid and 400-mg qd doses produced nearly complete IPA on day 1 and at day 28 (∼85–95%, Figure 4A), with the magnitude of inhibition at these doses being greater than that with the 50-mg bid dose of ticagrelor or with clopidogrel and with less variability in IPA (Figure 4B) [50]. Onset of maximum IPA effect was rapid and corresponded with plasma concentrations. Reversibility of effect was indicated by declining IPA values over the 24 h following the last dose (day 28). At 24 h after the last dose of study drug, ticagrelor at doses of 100 mg and higher maintained higher levels of IPA than clopidogrel.

### Safety/Tolerability

Ticagrelor treatment was well tolerated. Bleeding was the most frequently observed adverse event (Table 5); all bleeding events, defined according to a set of definitions modified from Clopidogrel in Unstable angina to prevent Recurrent Events (CURE) criteria, were minor and considered mild to moderate in severity, except for one major bleeding event in a patient receiving ticagrelor 400 mg qd. The incidence of bleeding appeared to increase with increasing ticagrelor dose. Dyspnea was also observed in ticagrelor patients, which appeared to be transient and dose-related. Of 29 cases of dyspnea (23 in patients treated with ticagrelor), 21 were considered mild and 8 moderate, and none was associated with congestive heart failure or bronchospasm. Overall, dyspnea lasted for up to 4 h in 6 of the patients, for 1 week or less in 5 more patients, for up to 2 weeks in 4 more patients, for up to 3 weeks in 3 more patients, and for more than 3 weeks in 5 more patients. Other adverse events occurring in at least 10% of a treatment group were dizziness and headache.

**Table 5:** Adverse events occurring in ≥10% in any treatment group in patients in DISPERSE

	Number (%) Ticagrelor 50 mg bid (n = 41)	100 mg bid (n = 39)	200 mg bid (n = 37)	400 mg qd (n = 46)	Clopidogrel 75 mg qd (n = 37)
Major bleeding events*	0 (0)	0 (0)	0 (0)	1 (2)	0 (0)
Minor bleeding events*	12 (29)	17 (44)	19 (51)	22 (48)	12 (32)
Venipuncture-site bruise	1 (2)	0 (0)	1 (3)	2 (4)	4 (11)
Epistaxis	1 (2)	4 (10)	4 (11)	8 (17)	2 (5)
Contusion	5 (12)	9 (23)	9 (24)	12 (26)	8 (22)
Red blood cells in urine	3 (7)	0 (0)	4 (11)	0 (0)	1 (3)
Dyspnea	4 (10)	4 (10)	6 (16)	9 (20)	0 (0)
Dizziness	4 (10)	2 (5)	1 (3)	4 (9)	1 (3)
Headache	0 (0)	5 (13)	1 (3)	1 (2)	3 (8)

From Husted et al. [50].

* A major bleeding event was defined as one that occurred in a critical site, was clinically overt and led to the transfusion of ≥2 units of packed red cells or whole blood, was clinically overt and associated with a fall in hemoglobin ≥20 g/L, or was fatal; all other bleeding events were classified as minor.

## DISPERSE-2: Non-ST-Elevation ACS Patients

In DISPERSE-2, 990 patients (984 in safety cohort) with non-ST-elevation ACS receiving aspirin and standard therapy for ACS were randomized to ticagrelor 90 mg bid (n = 334), with a profile similar to ticagrelor 100 mg bid studied in DISPERSE, or 180 mg (n = 323) bid or to clopidogrel (n = 327) at a 300-mg loading dose followed by 75 mg/day for up to 12 weeks [51]. Patients in the ticagrelor group were also randomized to a loading dose of 270 mg or no loading dose, and patients undergoing PCI within 48 h of randomization could receive an additional 300 mg of clopidogrel or matching placebo [52]; patients already receiving clopidogrel at study entry received clopidogrel 75 mg if randomized to the clopidogrel group. Across all treatment groups, 66% of patients underwent diagnostic coronary angiography, 43% had PCI, and 9% underwent coronary artery bypass grafting (CABG). The primary outcome measure was the Kaplan–Meier rate of major plus minor bleeding through 4 weeks.

### Bleeding

**Figure 5:**
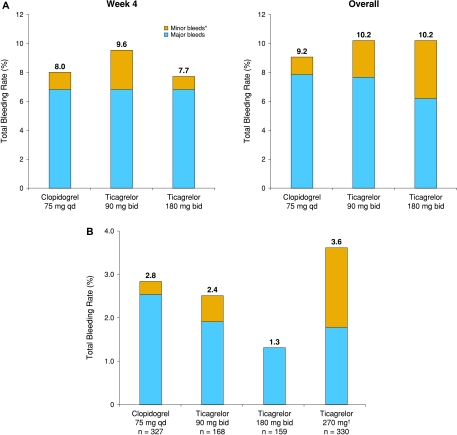
(**A**) Total bleeding rates through week 4 (left) and overall (right) in DISPERSE-2. (**B**) Bleeding rates within 48 h of randomization. *Minor bleeding without major bleeding; ^†^loading dose. Adapted with permission from Cannon et al. [51].

The Kaplan–Meier rate of protocol-defined major and minor bleeding (modified from CURE criteria; Table 6) did not differ significantly among treatment groups at 4 weeks, with rates of 8.1% in the clopidogrel group versus 9.8% in the ticagrelor 90 mg group and 8.0% in the ticagrelor 180 mg group (Table 7), with no significant difference among groups in rates of major bleeding [51]. Similarly, no significant differences were observed for total bleeding at week 12, with Kaplan–Meier event rates of 9.9% with clopidogrel versus 10.9% with ticagrelor 90 mg bid and 11.4% with 180 mg bid. Through week 12, there was a significant increase in minor bleeding at the higher ticagrelor dose. The actual incidence of major and minor bleeding (which differs from Kaplan–Meier rates due to differences in follow-up duration among patients) is shown in Figure 5A. The rate of major and minor bleeding within 48 h of randomization was higher in ticagrelor patients receiving a loading dose than in those not receiving a loading dose (Figure 5B).

**Table 7:** Number of bleeding events and Kaplan–Meier event rate in DISPERSE-2

	Number of events (event rate) [*P*-value vs. clopidogrel]		
	Clopidogrel 75 mg qd (n = 327)	Ticagrelor 90 mg bid (n = 334)	Ticagrelor 180 mg bid (n = 323)
Through week 4			
Total	26 (8.1)	32 (9.8) [0.43]	25 (8.0) [0.96]
Major	22 (6.9)	23 (7.1) [0.91]	16 (5.1) [0.35]
Fatal/life threatening	14 (4.4)	11 (3.4) [0.53]	10 (3.2) [0.44]
Other	8 (2.5)	12 (3.7) [0.38]	6 (1.9) [0.61]
Minor	4 (1.3)	9 (2.7) [0.18]	12 (3.8) [0.0504]
Through week 12			
Total	30 (9.9)	34 (10.9) [0.62]	33 (11.4) [0.72]
Major	26 (8.7)	26 (8.6) [0.96]	20 (6.3) [0.32]
Fatal/life threatening	16 (5.4)	13 (4.5) [0.55]	14 (4.3) [0.61]
Other	10 (3.3)	13 (4.2) [0.54]	6 (1.9) [0.34]
Minor	4 (1.3)	9 (2.7) [0.18]	16 (6.1) [0.01]

From Cannon et al. [51].

**Table 6:** Bleeding definitions in DISPERSE-2 (modified from CURE definitions)

Term		Associated with a ↓ in hemoglobin	Transfusion of whole blood or PRBCs for bleeding
Major bleed—fatal/life threatening	Fatal or intracranial or intrapericardial with cardiac tamponade or leading to hypovolemic shock or severe hypotension requiring pressors or surgery	>50 g/L (3.1 mmol/L)	≥4 units
Major bleed—other	Significantly disabling (e.g., intraocular with permanent vision loss)	30–50 g/L (1.9–3.1 mmol/L)	2–3 units
Minor bleed	Requires medical intervention (e.g., epistaxis requiring visit to medical facility for packing)		1 unit
Minimal bleed	All others not requiring intervention or treatment (e.g., bruising, bleeding gums, and oozing from injection site)		

From Cannon et al. [51].

PRBCs, packed red blood cells

Analysis of bleeding rates in patients undergoing CABG suggested the potential for clinical benefit of reversibility of the effect of ticagrelor in the setting of invasive intervention [51]. Among 84 patients undergoing CABG, median times to surgery from the last dose of study drug were 96 h among clopidogrel patients, 55 h among ticagrelor 90 mg bid patients, and 60 h among ticagrelor 180 mg bid patients. In patients undergoing CABG at 1–5 days after the last dose, major bleeding occurred in 36% (10 of 28) patients receiving ticagrelor, compared with 64% (9 of 14) receiving clopidogrel. Major bleeding occurred in 1 (50%) of 2 clopidogrel patients and 5 (50%) of 10 ticagrelor patients undergoing CABG within 1 day and in 6 (60%) of 10 clopidogrel patients and 10 (50%) of 20 ticagrelor patients undergoing CABG at more than 5 days.

### IPA

**Figure 6:**
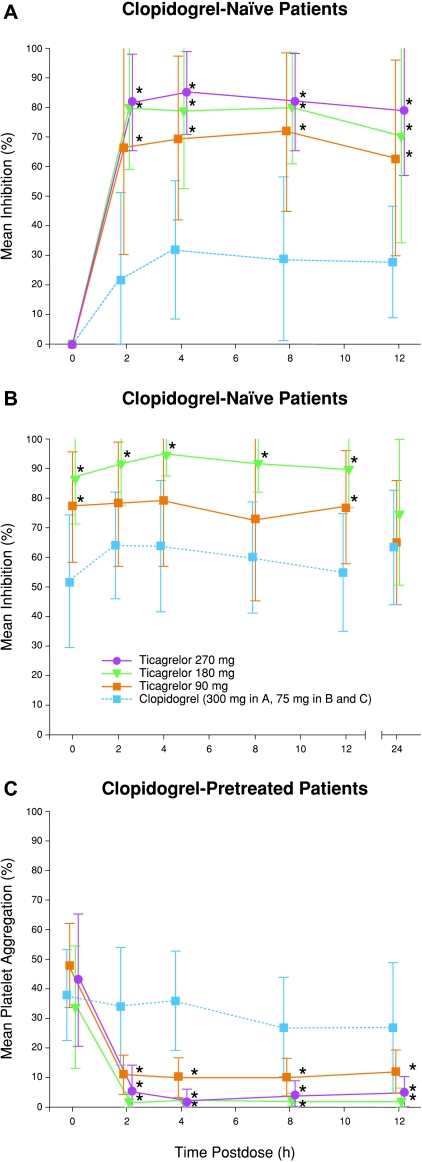
(**A**) Mean IPA (20 μM ADP, final extent) after first dose of ticagrelor 90 mg (n = 9) or 180 mg (n = 7) or 270-mg loading dose (n = 15) or of clopidogrel 300-mg loading dose (n = 14) in clopidogrel-naïve patients in DISPERSE-2. (**B**) IPA after 4 weeks of treatment and at 24 h after the end of treatment in clopidogrel-naïve patients receiving ticagrelor 90 mg bid (n = 15) or 180 mg bid (n = 10) or clopidogrel 75 mg/day (n = 10). (**C)** Change in percent of platelet aggregation among clopidogrel-pretreated patients receiving first dose of ticagrelor 90 mg (n = 9) or 180 mg (n = 7) or 270-mg loading dose (n = 16) or clopidogrel 75 mg (n = 12). Error bars indicate standard deviation, shown only for IPA sample space. *P <0.05 for ticagrelor versus clopidogrel. Adapted with permission from Storey et al. [52].

A pharmacodynamics substudy was performed in 45 clopidogrel-naïve patients and 44 clopidogrel-pretreated patients [52]. Among patients who had not received clopidogrel pretreatment, ticagrelor patients (n = 18 receiving 90 mg bid, n = 9 with loading dose; n = 13 receiving 180 mg bid, n = 6 with loading dose) had significantly greater IPA (final extent; 20 μM ADP) than clopidogrel patients (n = 14, all with loading dose) on day 1 (Figure 6A) [52]. IPA in ticagrelor-treated patients remained stable at 4 weeks and higher than that observed in clopidogrel-treated patients (Figure 6B). Among patients receiving clopidogrel prior to study treatment, the mean level of platelet aggregation at baseline in those receiving study clopidogrel 75 mg/day was 38% and did not change significantly (36%) at 4-h postdose testing (Figure 6C). In contrast, each of the ticagrelor doses produced a significant and substantial further reduction in platelet aggregation irrespective of baseline aggregation response (Figure 6C). Overall, ticagrelor treatment reduced residual platelet aggregation from 62.7 to 7.4% in those clopidogrel-pretreated patients in the highest tertile of baseline aggregation response and from 19.3 to 2.2% in those in the lowest tertile of baseline aggregation response.

### Pharmacokinetics

In a substudy in patients receiving ticagrelor 90, 180, or 270 mg (along with aspirin), mean plasma levels of ticagrelor were highest at the 2-h measurement after dosing and those of AR-C124910XX peaked 2–4 h after dosing and were one-fifth to one-half as high as ticagrelor [52].

### Safety/Tolerability

Adverse events are shown in Table 8[51]. Dyspnea appeared to be dose-related in ticagrelor patients, with a significantly greater frequency in the higher-dose group than in the clopidogrel group. Among all patients reporting dyspnea, 27% had resolution within 24 h and 48% (overall 2% of clopidogrel patients and 6% of each ticagrelor group) had symptoms lasting more than 15 days. As in DISPERSE, dyspnea episodes were mild or moderate, were frequently self-limiting, and infrequently led to drug discontinuation. There were no reported cases of fluid retention, congestive heart failure, or bronchospasm in ticagrelor patients reporting dyspnea, suggesting that the symptom is not related to heart failure.

**Table 8:** Adverse events in DISPERSE-2

	Number (%) of patients [*P*-value vs. clopidogrel]		
	Clopidogrel 75 mg qd (n = 327)	Ticagrelor 90 mg bid (n = 334)	Ticagrelor 180 mg bid (n = 323)
Dyspnea	21 (6.4)	35 (10.5) [0.07]	51 (15.8) [<0.0002]
Chest pain	29 (8.9)	25 (7.5) [0.57]	24 (7.4) [0.57]
Headache	28 (8.6)	32 (9.6) [0.69]	21 (6.5) [0.37]
Nausea	11 (3.4)	22 (6.6) [0.07]	21 (6.5) [0.07]
Dyspepsia	9 (2.8)	16 (4.8) [0.22]	10 (3.1) [0.82]
Insomnia	9 (2.8)	18 (5.4) [0.12]	15 (4.6) [0.22]
Diarrhea	11 (3.4)	10 (3.0) [0.83]	24 (7.4) [0.02]
Hypotension	2 (0.6)	4 (1.2) [0.004]	12 (3.7) [0.01]
Dizziness	10 (3.1)	14 (4.2) [0.53]	11 (3.4) [0.83]
Syncope	2 (0.6)	4 (1.2) [0.69]	5 (1.5) [0.28]
Rash	2 (0.6)	3 (0.9) [1.00]	6 (1.9) [0.17]

From Cannon et al. [51].

The rates are crude incidences of number of patients with reported events divided by the total number of patients in the safety cohort.

Hypotension was also more common in ticagrelor groups, occurring in 1.2% of ticagrelor 90 mg bid patients: 3.7% of ticagrelor 180 mg bid patients and 0.6% of clopidogrel 75 mg qd patients. Overall discontinuation rates were low and similar between groups: 6% of ticagrelor 90 mg bid patients; 7% of patients receiving 180 mg bid; and 6% of patients receiving clopidogrel 75 mg qd. Analysis of continuous ECG monitoring during the first 4–7 days of treatment in 885 patients indicated that there was no significant difference among groups in rates of ventricular tachycardias. However, a *post hoc* analysis showed a greater frequency of mostly asymptomatic ventricular pauses persisting for more than 2.5 seconds among ticagrelor patients, with rates of 4.3% among clopidogrel patients, 5.5% (*P*= 0.58) among ticagrelor 90 mg bid patients, and 9.9% (*P*= 0.014) among ticagrelor 180 mg bid patients. The proportions of patients with more than three episodes were 0.3, 2.0 (*P*= 0.12), and 4.9% (*P* <0.001), respectively, and the proportions of those with at least one episode of more than 5 seconds were 0.3, 1.6 (*P*= 0.22), and 2.1% (*P*= 0.06). However, the observed pauses did not lead to discontinuation of study drug and were not associated with clinical symptoms such as dizziness or syncope.

The mechanisms for dyspnea and ventricular pauses are unknown and currently being investigated. Although ticagrelor is not an ATP or adenosine analog, it is possible that it affects adenosine metabolism, as is the case with a number of agents that affect cellular adenosine levels by interfering with adenosine degradation and reuptake. Such an effect could account for the increased incidence of ventricular pauses and dyspnea [51].

### Cardiovascular Events

There were numerically fewer myocardial infarctions over 12 weeks with ticagrelor treatment, with Kaplan–Meier event rates of 5.6% in the clopidogrel group versus 3.8% in the ticagrelor 90 mg bid group and 2.5% in the ticagrelor 180 mg bid group [51].

## Phase III PLATO Trial

PLATelet Inhibition and Patient Outcomes (PLATO) is an international, randomized, double-blind, double-dummy phase III trial examining the safety and efficacy of ticagrelor compared with clopidogrel in approximately 18,000 patients with either non-ST-elevation ACS or ST-elevation ACS [53]. This trial is designed to test the hypothesis that ticagrelor compared with clopidogrel on a background of aspirin will result in a lower risk of recurrent ischemic events in a broad patient population with ACS in a setting designed to reflect current and evolving medical practice.

Patients are randomized to receive ticagrelor at a loading dose of 180 mg followed by 90 mg bid or clopidogrel at a loading dose of 300 mg, with provision for an additional 300 mg in patients undergoing PCI, followed by 75 mg qd for up to 12 months. Patients already receiving clopidogrel at study entry are to receive a ticagrelor loading dose or matching placebo, followed by the daily regimen of either ticagrelor or clopidogrel. All patients are to receive aspirin (75–100 mg/day). The primary efficacy outcome is the composite endpoint of death from vascular causes, myocardial infarction, or stroke. The primary safety outcome is PLATO-defined and -adjudicated major bleeding; the criteria used in PLATO were identified as constituting the most appropriate and clinically meaningful assessment of bleeding complications associated with chronic therapy and are based on those used in CURE (Table 9). Efficacy and safety outcomes are to be assessed both overall and separately in patients with non-ST-elevation ACS and patients with ST-elevation ACS, and bleeding outcomes are to be assessed in relation to timing of PCI and CABG. A program for assessing the occurrence of arrhythmic events in which a subpopulation of patients underwent Holter monitoring for 7 days after randomization (n = 2900) and again at 1 month (n = 2000) was completed in November 2007.

**Table 9:** PLATO bleeding definitions* (modified from CURE definitions)

Term		Associated with a ↓ in hemoglobin	Transfusion of whole blood or PRBCs for bleeding
Major bleed—life threatening	Fatal or intracranial or intrapericardial with cardiac tamponade or hypovolemic shock or severe hypotension requiring pressors or surgery	>50 g/L (3.1 mmol/L)	≥4 units
Major bleed—other	Significantly disabling (e.g., intraocular with permanent vision loss)	30–50 g/L (1.9–3.1 mmol/L)	2–3 units
Minor bleed	Requires medical intervention to stop or treat bleeding		
Minimal bleed	All others not requiring intervention or treatment		

From James et al. [53].

PRBCs, packed red blood cells.

*If the bleeding event fulfills criteria in more than one category, the event will be assigned to the most severe category.

Study recruitment for PLATO ended in July 2008, with enrollment of over 18,000 patients.

## Conclusion

Ticagrelor, a CPTP agent, is the first reversibly binding oral P2Y_12_ receptor antagonist. It produces plasma concentration-dependent IPA that is rapid in onset and greater in magnitude and less variable than that observed with clopidogrel. The pharmacologic and clinical profiles of this agent suggest that it may be capable of providing a high and consistent level of antithrombotic protection without a proportional increase in bleeding risk and will likely offer more rapid offset of effect than do current thienopyridine P2Y_12_ inhibitors. These characteristics may improve the overall efficacy of antiplatelet therapy and facilitate management of ACS patients, including those who will or may require surgical intervention and thus would be at increased bleeding risk. Additionally, ticagrelor shows a similar safety and tolerability profile to clopidogrel in phase II trials and thus may provide a safe alternative to current therapy. The PLATO trial will provide information on safety and efficacy of ticagrelor in a broad spectrum of ACS patients.
